# Venom immunotherapy and difficulties encountered before and during immunotherapy: Double sensitization, systemic reactions, treatment with omalizumab, and high dose VIT

**DOI:** 10.55730/1300-0144.5427

**Published:** 2022-04-10

**Authors:** Gülden PAÇACI ÇETİN, İnsu YILMAZ, Murat TÜRK, Bahar ARSLAN, Sakine NAZİK BAHÇECİOĞLU

**Affiliations:** 1 Division of Immunology and Allergy, Department of Chest Diseases, Faculty of Medicine Erciyes University, Kayseri, Turkey; 2 Division of Immunology and Allergy, Department of Chest Diseases, Kayseri City Training and Research Hospital, Kayseri, Turkey; 3 Division of Immunology and Allergy, Department of Chest Diseases Atatürk Chest Disease and Thoracic Surgery Training and Research Hospital, Ankara, Turkey

**Keywords:** Allergy, venom immunotherapy, Apis Mellifera, Vespula, double sensitization, omalizumab

## Abstract

**Background/aim:**

Venom immunotherapy (VIT) is the most effective treatment method to prevent recurrent systemic reactions to Hymenoptera stings. In this study, the demographic characteristics of VIT patients, the success rates of VIT, the difficulties we encountered during VIT, and solutions for these difficulties in our clinic were presented.

**Materials and methods:**

We retrospectively analyzed patients with venom allergy who applied venom immunotherapy between 2013–2020. Data on age, gender, Hymenoptera species with the first reaction, grade of the reaction, beekeeping history, skin prick and specific IgE and component results, double sensitization, blood groups, and reactions with VIT and/or sting during built-up and maintenance periods were recorded.

**Results:**

A total of 73 patients were enrolled in the study. The median time from the first sting reaction to the application to the allergy outpatient clinic was 12 (0.5–24) months. The first sting reaction of 38 (52.1%) of the patients was with honey bees, and 24 (32.9%) were with wasps. Double positivity was present in 29 (40%) of the patients in prick results and 26 (36%) serologically. There was no correlation between the severity of first reactions and Apis Mellifera or Vespula prick diameters (p = 0.643; r = −0.056; p = 0.462; r = 0.089, respectively). High-dose VIT was administered to 4 patients. Omalizumab has been used as an alternative agent to achieve the maintenance dose in 2 patients with frequent systemic reactions during VIT.

**Conclusion:**

Most patients were able to tolerate VIT. Double positivity is one of the most common difficulties before VIT. In patients who develop systemic reactions in the VIT maintenance phase, a maintenance dose increase should be considered in the maintenance phase. Adding omalizumab does not seem to be a permanent solution in patients who develop a severe systemic reaction.

## 1. Introduction

A Hymenoptera sting is a common condition in society, and 56%–94% of people are stung at least once in their lifetime [1]. Allergic reactions to Hymenoptera venom are often extensive local reactions at the injection site and systemic reactions. Large local reaction (LLR) was defined as painful swelling and erythema exceeding 10 cm in diameter lasting longer than 24 h, limited to the skin and subcutaneous tissue [2]. Systemic sting reactions (SSR) may occur with multiple organ involvement and anaphylaxis; it can also be seen in milder forms such as urticaria and/or angioedema [3]. The prevalence of systemic reactions in European epidemiological studies is between 0.3% and 7.5% in adults, and broad local reactions are 2%–4%–26.4% in the population [4, 5]. Severe reactions can be life-threatening and mortal. Patients diagnosed with venom allergy should carry emergency kits containing adrenaline auto-injector, H1 antihistamines, and corticosteroids against the risk of anaphylaxis.

Venom immunotherapy (VIT) is the most effective treatment method to prevent recurrent systemic reactions [6, 7]. It is effective in 77%–84% of the patients undergoing immunotherapy (IT) with honey bee venom and in 91%–96% of patients receiving immunotherapy with wasp venom [8]. Although VIT success is high, some difficulties may be encountered before IT and in the IT process. Detection of SPT (skin prick test) and/or specific immunoglobulin E (sIgE) double positivity against different species, especially in persons stung with unknown bee species, leads the clinician to make further examinations and make the decision more carefully when selecting the proper venom extract [9–11]. Another difficulty is serious systemic reactions that can occur during the build-up and maintenance doses of IT. VIT is restarted with dose reduction, especially in severe systemic reactions seen with VIT during the build-up phase. Still, despite this, VIT can be continued with omalizumab to suppress the secondary systemic effects related to VIT and continue IT in patients who have anaphylaxis after VIT. In cases with severe anaphylaxis in the maintenance phase, high-dose VIT should be considered.

In this study, the demographic characteristics of VIT patients, the success rates of VIT, and the difficulties we encountered during VIT in our clinic were discussed together with the current literature.

## 2. Materials and methods

This study was conducted by examining the data of 2013–2020 in Erciyes University Immunology and Allergy Diseases Clinic, retrospectively. The data of patients who had an SSR after a honey bee or wasp sting and who started VIT in line with the guideline recommendations were analyzed. Data on age, gender, bee species with the first reaction, grade of the reaction, beekeeping history, skin prick and specific IgE and component results, blood groups, and reactions with IT and/or bee sting during built-up and maintenance periods were recorded.

In our clinic, conventional VIT was applied to all patients with the same allergen extract (Alutard^®^ SQ, ALK-Abelló, Denmark). The maintenance phase was reached at approximately 16 weeks. However, in patients who experienced a systemic reaction in the build-up phase, the time to reach the maintenance phase was prolonged, since step-downs were made according to the severity of the reaction. At the end of the second year, the dose intervals were increased to 6 weeks, and 8 weeks after 3 years, in patients who did not experience any systemic reactions at the beginning and maintenance and we thought that there would be no compliance problems. The built-up phase was started with 20 U-SQ/mL, and the dose was increased up to the standard maximum maintenance dose of 100,000 U-SQ/mL. Routine use of antihistamines was recommended as a premedication before VIT in the built-up phase. All doses were administered in the equipped immunotherapy room under the supervision of a doctor. In cases where the systemic reaction was observed in the built-up phase, a dose reduction was made according to the reaction intensity, and it was restarted from the recommended previous doses. VIT was continued along with omalizumab treatment in some of the patients whose systemic reaction continued. In the case of a systemic reaction with VIT and/or bee sting in the maintenance phase, the dose was reduced by 2–4 steps, and the maintenance dose was increased up to 150,000 U-SQ/mL. Underlying factors such as double-sensitization, high tryptase levels, and mastocytosis that may affect the success of VIT were investigated in all patients.

Ring and Messmer Anaphylaxis Grading Scale were used to grade the reaction [12].

### 2.1. Skin prick test (SPT)

**Figure 1:**
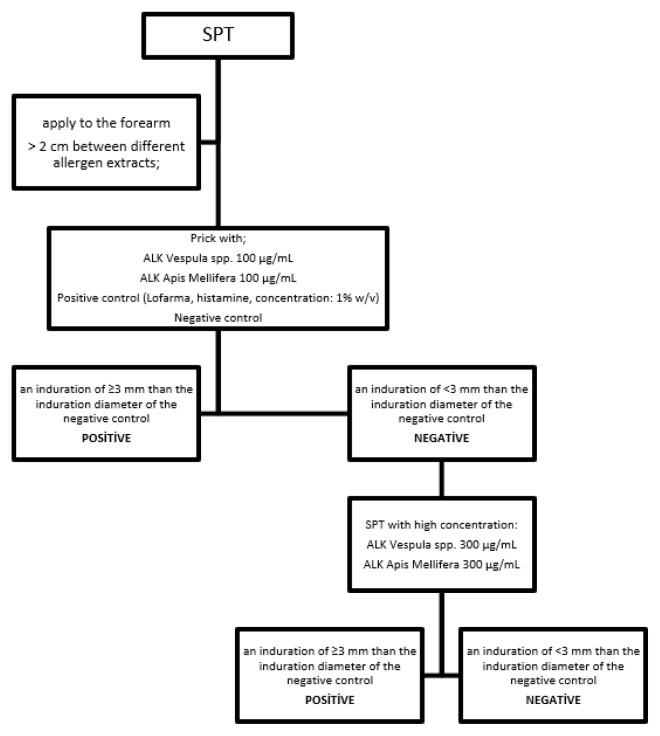
A schematization for SPT. SPT: Skin prick test

Allergen extract drops were first applied to the forearm. This was followed by pricking the skin with a special lancet (Heinz Herenz Hamburg, Germany). A distance of more than 2 cm was ensured between different allergen extracts. At the end of 20 min, the induration size that was ≥3 mm larger than the size of the induration resulting from a negative control was considered positive. Apis Mellifera and Vespula SPT extract (ALK Vespula spp. 100 μg/mL, ALK Apis Mellifera 100 μg/mL, ALK Vespula spp. 300 μg/mL, ALK Apis Mellifera 300 μg/mL) were used to conduct the test in all patients. Skin prick tests were performed with bee venom extracts at a concentration of 100 μg/mL, if it was negative, the bee venom extract concentration dose was increased to 300 μg/mL (Figure 1). Before starting VIT in patients who developed a systemic reaction with skin prick test at these concentrations, VIT was started with lower concentrations than the current starting dose. The test was performed by an experienced nurse and doctor at the clinic. All patients had vascular access before SPT.

### 2.2. Sample measurement

The serum Apis Mellifera specific IgE and Vespula-specific IgE levels were determined with the ELISA method (ThermoFisher Scientific ImmunoCAP, U.S.). Classification in this method is class 0: < 0.35 kU/L (no allergy), class 1: 0.35–0.7 kU/L (low positive), class 2: 0.70–3.5 kU/L (positive), class 3: 3.50–17.5 kU/L (strong positive), class 4: 17.5–50 kU/L (high positive), class 5: 50–100 kU/L (very high positive), and class 6: ≥100 kU/L (extremely positive)[13].

### 2.3. Statistical analysis

The data analysis was performed by SPSS statistical software (SPSS Inc. Chicago, Illinois) version 22. The central tendency and dispersion of numerical data were shown as mean ± standard deviation (SD) for normally distributed data and median (interquartile range) for the nonnormally distributed data. The analysis of normally distributed data was performed with the Kolmogorov Smirnov test. Comparisons between independent groups were performed with Pearson chi-square test for categorical variables and with independent sample t-test, Mann Whitney U test or Kruskal Wallis for continuous variables. Spearman’s correlation was used to detect the relationship between parameters. P-value < 0.05 was considered significant.

## 3. Results

The general characteristics of 73 patients who underwent VIT are shown in Table 1. Thirty-eight (52%) of the patients were female, and the mean age of all patients was 43 ± 12.9 years. Seventeen (23.3%) patients were dealing with beekeeping either professionally or as a hobby. The first sting reaction of 38 (52.1%) of the patients was with honey bees, and 24 (32.9%) were with wasps. Eleven (15.1%) patients did not know the type of Hymenoptera with which the first reaction was experienced. The median time from the first sting reaction to the application to the allergy outpatient clinic was 12 (0.5–24) months. The reaction severity was made according to the Ring and Messmer classification; a mild reaction was observed in 6 (8.2%) of the patients, while 67 (91.2%) experienced a moderate and severe reaction. At least one of the pricks and/or specific IgE results of all patients had a positive Apis Mellifera or Vespula. Double positivity was present in 29 (40%) of the patients in prick results, in 26 (36%) of the patients serologically, and in 41 (56%) of the patients in at least one of these results.

Since beekeeping constitutes the risk group for honey bee anaphylaxis, the characteristics of those engaged in beekeeping and those who do not have been compared. The general characteristics of the patients engaged in beekeeping as a hobby or professionally and those who do not are shown in Table 2. The first reaction of 15 (88%) of 17 patients who are beekeeping developed against honey bees. Four out of 6 patients who started VIT with Grade 1 reaction were beekeepers. Although the time from the first sting reaction to the application to the outpatient clinic was longer in beekeepers than nonbeekeepers, the difference was not statistically significant (12 [0.25–24] vs. 10.5 [0.5–24]; p = 0.85). Apis Mellifera positivity was significantly higher in prick (p = 0.001), and specific IgE (p = 0.005) results in beekeepers. The double-positivity rate was higher in beekeepers, and a significant difference was found between those who are nonbeekeepers (77% vs. 50%; p = 0.042).

In 62 patients who knew the culprit insect in the first reaction, allergic sensitivity was demonstrated by at least one method and immunotherapy was initiated with the same species. In 6 of 11 patients who did not know the type of the culprit insect, only sensitization to the Vespula was detected, and immunotherapy was started with the detected bee venom. Of the 5 patients who did not know the type of culprit insect, 3 had double sensitization with skin prick and mono sensitization with specific IgE. Immunotherapy was initiated in these patients with venom type positive for both skin prick and specific IgE (2 patients Vespula, 1 patient Apis Mellifera). Sensitivity to both bee venom with skin prick and specific IgE was detected in 2 patients. Since the Vespula had specific IgE 5+ (64 kU/L) and Apis Mellifera specific IgE 1 + (0.58 kU/L), immunotherapy was started with Vespula in one of these patients. Venom components were studied in the other patient. Immunotherapy with Vespula was started in the patient who was found to be Api m1: negative, Ves v1: negative, Ves v5: positive on component analysis of venom.

**Figure 2:**
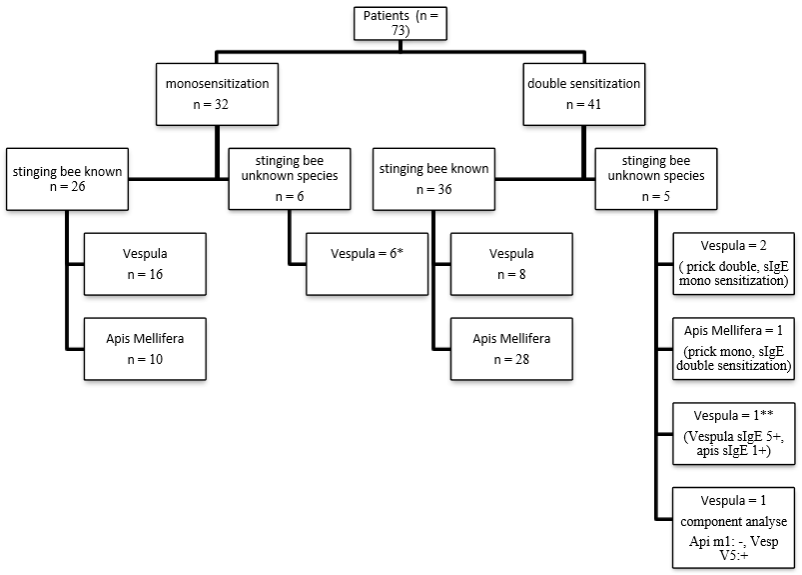
IT type of mono-sensitized and double-sensitized patients. * Monosensitization was detected in both skin prick and specific IgE in four of six patients sIgE: specific immunoglobulin E. One patient was monosensitize with spesific IgE and double negative with skin prick test. One patient was monosensitize with skin prick test and double negative with spesific IgE. ** vespula spesific IgE: 64 kU/L, apis mellifera spesific IgE: 0.58 kU/L

Venom components were studied because one patient had a history of reaction after a bee sting with both honey bee and Vespula and had double sensitization with the SPT. As Api m1: positive, Ves v1: positive, and Ves v5: positive on component analysis of venom, VIT was started with both venoms since sensitivity to both venom major allergens was detected (Figure 2).

Apis VIT was started for 38 patients, Vespula VIT was started for 34 patients, and 1 patient was started immunotherapy with both Apis Mellifera and Vespula. Six (15.7%) of 38 patients who started Apis VIT completed 5-year VIT, and 19 (50%) patients are continuing their immunotherapy. Three (0.08%) of 34 patients who started Vespula VIT completed 5 years of VIT, and 25 (73.5%) patients are continuing their immunotherapy. In 4 patients, VIT could not be achieved despite dose reduction and resumption of VIT due to built-up or maintenance phase reactions (3 patients for Apis VIT; 1 patient for Vespula VIT). In 15 patients, although the importance of VIT was explained, VIT was terminated according to the patients’ requests.

**Figure 3:**
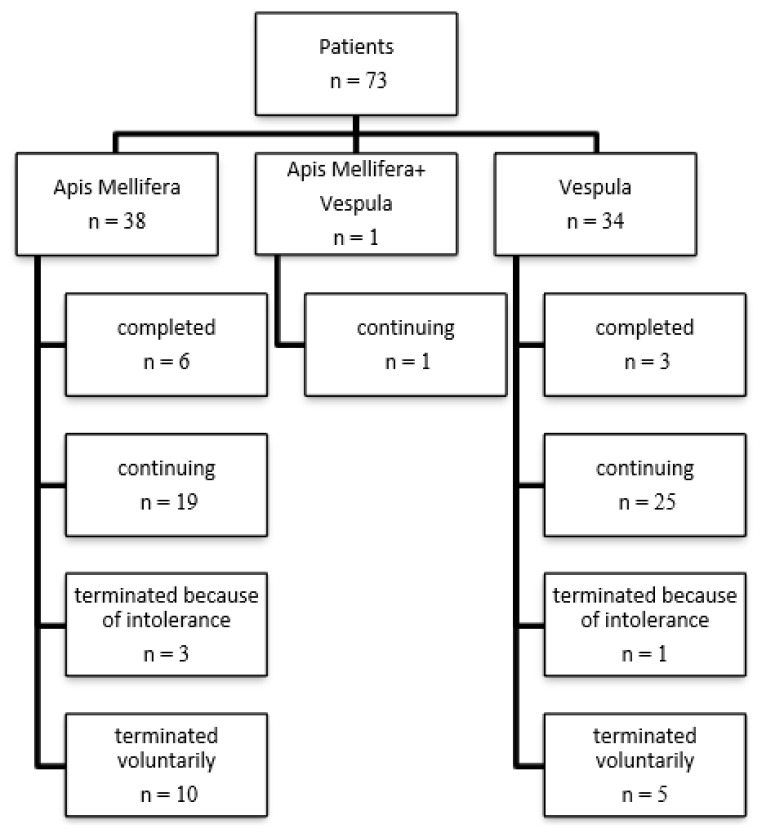
Treatment processes of patients receiving IT.

VITs of 44 patients who underwent Apis and Vespula VIT continue without any problem and have not yet completed 5 years (Figure 3).

### 3.1. Correlation between SPT and reaction severity

There was no correlation between the severity of the first reaction and the Apis Mellifera prick diameter in patients who underwent VIT (p = 0.643; r = −0.056). There was no correlation between the severity of the first reaction and the diameter of the Vespula prick in patients who underwent VIT (p = 0.462; r = 0.089).

### 3.2. Reaction severity across blood types

Blood groups of 66 of the patients who received VIT were taken. The blood type of 31 patients was A, the blood type of 9 patients was B, the blood type of 5 patients was AB, and the blood type of 21 patients was 0. Sixty patients were Rh +, 6 patients were Rh -.

There was no difference between ABO blood groups in terms of reaction severity (p = 0.394) (Table 3). There was no difference in reaction severity between Rh blood groups (p = 0.533) (Table 4).

### 3.3. VIT and systemic reactions

**Figure 4:**
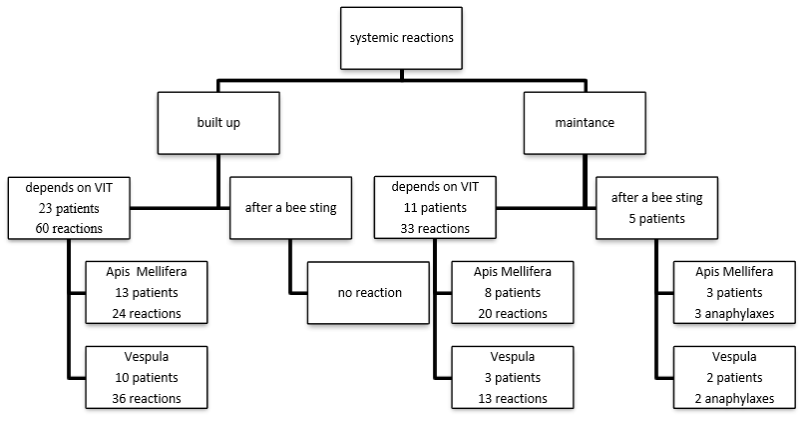
IT-related systemic reactions. VIT: venom immunotherapy

All patients to whom we applied VIT received a single dose of antihistamine 1 h before VIT in the build-up phase. During the build-up phase, 60 systemic reactions due to VIT were observed in 23 different patients. Of these 23 patients, 13 patients had immunotherapy with Apis Mellifera (24 reactions in total) and 10 patients with Vespula (36 reactions in total). In the built-up phase, no reaction was observed in any of the 5 patients stung by the bee species who were administered immunotherapy. During the maintenance phase, a total of 33 systemic reactions were observed in 11 different patients. Of these 11 patients, 8 patients were in the Apis Mellifera (20 reactions) and 3 patients were in the Vespula (13 reactions) group. The most common systemic reactions are dyspnea and numbness in the hand and foot. Other systemic reactions are nausea, palpitations, dizziness, pruritus, uvula edema, and hypotension, respectively. The demographic and characteristic data of these patients are summarized in Table 5. Two of these patients had high levels of tryptase, none of them have any autoimmune disease, only one patient used beta-blocker, seven patients were polysensitized. In the maintenance phase, no reaction was observed in 13 of 18 patients who were injected with bees, who received immunotherapy, and 5 patients who developed anaphylaxis (Figure 4).

### 3.4. Omalizumab and VIT

VIT was continued with omalizumab treatment in 2 patients who experienced a systemic reaction during the bee VIT build-up and could not tolerate VIT during the step-up process after dose reduction. Immunotherapy of the first patient was discontinued due to continuing systemic reactions despite dose increase under omalizumab treatment. The patient did not accept further diagnostic tests for mastocytosis. IT was applied under omalizumab due to the development of anaphylaxis during the dose increase in the other patient. In this way, the immunotherapy of the patient could be increased to the maintenance phase dose. Anaphylaxis developed at the third maintenance dose in the patient who received two maintenance doses without any problem without omalizumab. IT was discontinued when it was decided that the desired immunomodulatory efficacy could not be achieved due to the systemic reaction at the maintenance dose after omalizumab was discontinued [14]. Venom immunotherapy protocols administered to the patient with omalizumab are summarized in Table 6.

### 3.5. High dose VIT

High dose VIT was given with Apis Mellifera in 3 patients and Vespula in 1 patient (150,000 U), as a systemic reaction developed during VIT during the maintenance or the systemic reactions after a bee sting with the bee species in which VIT was performed during the maintenance. While three of these patients continued with the VIT without any problem, VIT of one patient was terminated due to frequent reactions with high doses.

## 4. Discussion

In our study, it was seen that Apis Mellifera VIT was the majority of those who had VIT, and those who had a history of systemic reactions with bees had a long time between the application of bee VIT. Double positive sensitization was detected in DPT and/or sIgE in about half of the patients. There was no correlation between skin prick, specific IgE, and blood types of VIT patients and their reaction severity in the history. The most common systemic reaction was experienced with the Vespula VIT. It was observed that the use of omalizumab together in patients who could not tolerate VIT was not very effective in immune switching. High-dose VIT could be tolerated. With this study, the characteristics of our patient cohort receiving bee VIT and the difficulties and solutions encountered before and during VIT were revealed.

The family Apidae consists of the Apis mellifera (honeybees) and the Bombus (bumble-bees) species. The family Vespidae consists of the Vespinae [three genera: Vespa (hornets), Dolicho-Vespula (wasps), Vespula (wasps or yellow jackets)] and the Polistinae (single genus: Polistes, wasps) subfamilies [4]. Sting reactions in the entire Mediterranean area are most frequently caused by Vespula, Polistes, and Apis Mellifera. In our study, VIT was applied more with Apis Mellifera. Excessive beekeeping in our country and our region causes us to encounter more systemic reactions seen with honey bees. Before VIT is initiated in a person with a history of systemic reaction with bees, sensitization must be demonstrated by at least one skin prick, specific IgE, and/or basophil activation tests. In our study, it has been shown that the time from systemic reaction to sensitization with bees is very long. Although all of our patients applied to the emergency department after these reactions, their application to the allergy outpatient clinic was too late, and recurrent stings might occur during this time. In our patient group, this period was longer in beekeepers than in nonbeekeepers. For this reason, we think that social awareness should be raised to increase awareness on this issue to make these periods earlier.

Sensitization to both Apis Mellifera and Vespula venom is common in people with insect venom allergies. It is difficult to determine whether this is due to true double sensitization or cross-reactivity. It is essential to choose the proper venom preparation to get the desired benefit from VIT. Double positivity is common between Vespula and Apis Mellifera in patients who experience systemic allergic reactions after a bee sting [15, 16]. The major cross-reactive component between these two groups is hyaluronidase [4, 17, 18]. The diagnostic tests available are inadequate to distinguish between asymptomatic sensitization and clinically significant allergy [3]. There is no common consensus on the continuation of IT single or double in individuals with dual sensitization. In our study, 56% of patients had double sensitization. This ratio is between 30%–59% in previous studies [10, 19]. Regarding the subject, in the algorithm developed by Johanna et al., in cases where the culprit insect is known, even if double sensitization is detected in skin prick and/or serological test results, a single VIT can be performed with the culprit type. In cases where the culprit insect is unknown, if there is a significant difference between the skin prick and serological tests and sensitization against a single venom is stronger, it recommends performing a single VIT again [20]. In our clinic, similar to the algorithm developed by Johanna et al., in cases where the culprit insect is known, we do a single VIT with the culprit type even if double sensitization is detected in the skin prick and/or serological test results. In cases where the culprit insect is not known, if there is a significant difference between skin prick and serological tests and sensitization against single venom is stronger, we do single VIT again. However, in cases where the culprit insect is unknown, we work with components if there is no significant difference between the skin prick and serological tests. Whichever bee species-specific major component is positive, we apply VIT to patients with that species. If the major component of both bee species is positive, we apply VIT to both of them.

Another issue that is curious about bee venom allergies is whether there is a relationship between SPT and/or specific IgE results and the severity of the systemic reaction. In a study by Annila et al., no relationship was found between the degree of systemic reaction experienced in the past in beekeepers and the serum levels of Apis Mellifera specific IgE antibodies. SPT with Apis Mellifera was significantly higher in 31 patients who had systemic reactions than beekeepers who had local or no reactions (p < 0.05) [21]. In a study investigating risk factors for systemic reactions in patients with venom allergy, no significant relationship was found between the amount of both Vespula and Apis sIgE and SPT and the severity of systemic reactions [22]. In the study conducted by Warrington et al., no correlation was found between the severity of the clinical reaction and the degree of skin test reactivity or sIgE levels in the analysis of 36 patients with sudden hypersensitivity reactions to bee venom [23]. In our study, too, no correlation was found between the severity of systemic reactions and the results of SPT and sIgE. When we questioned whether there was a relationship between blood groups and reaction severity, we found no difference between AB0 blood groups in terms of reaction severity since the relationship had never been examined before.

VIT with the honey bee is an independent predictor for the high risk of systemic reactions during immunotherapy [24, 25]. In our study, in line with previous information, the number of patients who experienced systemic reactions was higher in the Apis Mellifera VIT group. Omalizumab has been used as an alternative agent to achieve the maintenance dose in patients with frequent systemic reactions during VIT [26, 27]. However, it was observed that the use of omalizumab together in patients who could not tolerate VIT was not very effective in immune switching. In a patient who experienced a systemic reaction during VIT dose increase, monthly omalizumab treatment was given 6 months before VIT, then VIT was restarted, but VIT could not be continued due to recurrent systemic reactions [28]. In our study, omalizumab was given to two patients who could not tolerate VIT, but these patients continued to have systemic reactions with VIT after omalizumab was discontinued [14]. This suggests that omalizumab has a strong premedication effect but not an immunomodulatory effect on VIT. Failure of VIT and omalizumab combination therapy may be caused by the lack of standardization regarding omalizumab dose, administration interval, administration time, and how long before VIT is restarted.

Increasing the dose with the rush and ultra-rush protocols may increase the chance of success in patients with maintenance reactions, but on the other hand, there is a potential to increase the risk of VIT side effects with these protocols. In addition, VIT cannot be performed in these protocols due to the lack of aqueous extracts in our country.

In some patients, the immune-modifying effect of VIT cannot be achieved with a routine 100 μg maintenance dose, and higher doses are required. The maintenance dose can be increased to 200 μg in patients who develop systemic allergic reactions after a bee sting or during VIT [3]. This dose can be well tolerated by patients [29]. However, in our clinical practice, we increase the dose up to 150 μg (150,000 U), if there is a systemic reaction with this dose, we plan to increase it to 200 μg (200,000 U). In our study, the high dose (150 μg) was increased in a total of four patients; although three of the patients tolerated it well and had no reaction, immunotherapy was discontinued due to the persistence of systemic reactions in one of them.

One of the study’s limitations is that our study is retrospective, and the number of patients is partially low. Especially the number of patients who developed reactions during VIT maintenance and switched to high-dose maintenance treatment and the number of patients who developed built-up systemic reactions and continued VIT with omalizumab is quite limited. However, we think that these results will contribute to clinicians’ management of patients in situations where these problems are encountered.

One of the limitations of the study is that it is difficult to rule out double sensitization because component-based tests were not performed in all patients. However, in patients with double sensitivity and whose history is unclear, the component was studied by explaining its importance to the patient. On the other hand, VIT was planned according to the anamnesis for double positivity, which clearly describes the stinger and the reaction (e.g., a beekeeper who knows bees well and knows that stung by honeybees).

A final limitation is the absence of all components to which the patient may be sensitized in venom immunotherapy extracts. One of the advantages of component-based tests is that it can be determined whether there is sensitivity to components that are not included in the venom IT contents. In cases where VIT fails, the components that are not included in the extracts can also be studied to reveal the reason for the failure.

In conclusion, although systemic reactions were seen more in Apis Mellifera VIT patients, most patients could tolerate VIT. Double positivity before VIT is one of the most common difficulties before immunotherapy. This problem can be solved with appropriate algorithms according to whether the suspected allergen is known or not, SPT/sIgE results, and components. One of the common difficulties in the VIT process is the systemic reactions that occur secondary to VIT. A maintenance dose increase should be considered for those who have reacted in the maintenance phase. However, adding omalizumab to VIT does not seem to be a permanent solution in patients who develop a built-up severe systemic reaction. VIT cannot be tolerated, although the dose is decreased and increased gradually.

## Figures and Tables

**Table 1:** Patients’ characteristics.

	Patients n = 73
**Age ± SD (years)**	43 ± 12.9
**Gender; female (%)**	38 (52)
**Culprit insect type in the first reaction; n (%)**	
Apis Mellifera	38 (52.1)
Vespula spp.	24 (32.9)
Unknown	11 (15.1)
**Time elapsed after the first reaction; median month (IQR)**	12 (0.5–24)
**First reaction grade; n (%)**	
Grade 1	6 (8.2)
Grade 2	19 (26)
Grade 3	28 (38.4)
Grade 4	20 (27.4)
**Beekeeping; n (%)**	17 (23.3)
**Prick A. Mellifera positivity; n (%)**	48 (65.7)
**Prick A. Mellifera; median longest diameter (IQR) (mm)**	3 (0–5)
**Prick Vespula positivity; n (%)**	51 (69.8)
**Prick Vespula; median longest diameter (IQR)mm**	3 (0–4)
**sIgE Apis positivity (n = 64) n (%)**	38 (59)
**sIgE Vespula positivity (n = 62) n (%)**	45 (72.5)
**Double-positives based on Prick results; n (%)**	29 (40)
**Double-positives based on sIgE result; n (%)**	26 (36)
**Double-positives by any method; n (%)**	41 (56)
**Blood types AB0 (n = 66)**	
A	31
B	9
AB	5
0	21
**Blood types Rh (n = 66)**	
Positive	60
Negative	6
**The type of IT; n (%)**	
Apis mellifera	38 (52.2)
Vespula spp	34 (46.5)
Apis mellifera + Vespula spp	1 (1.3)

sIgE: specific IgE, IT: immunotherapy, IQR: interquartile range

**Table 2:** General characteristics of patients with and without beekeeping.

	Beekeepers n = 17	Nonbeekeepers n = 56	P
**Age ± SD**	47.2 ± 10.3	41.8 ± 13.5	0.135
**Gender; female (%)**	4 (24)	34 (61)	0.007
**Culprit insect type in the first reaction (%)**			
Apis melifera	15 (88)	23 (41)	0.003
Vespula spp.	1 (6)	23 (41)	
Unknown	1 (6)	10 (18)	
**Time elapsed after first reaction; month (IQR)**	12 (0.25–24)	10.5 (0.5–24)	0.85
**First reaction grade; n (%)**			
Grade 1	4 (24)	2 (4)	0.043
Grade 2	2 (12)	17 (30)	
Grade 3	6 (35)	22 (39)	
Grade 4	5 (29)	15 (27)	
**Prick positivity; n (%)**			
Apis Mellifera	17 (100)	31 (55)	0.001
Vespula spp	9 (53)	42 (75)	0.115
**sIgE positivity; n (%)**			
Apis Mellifera	15 (88)	23 (41)	0.005
Vespula Spp	9 (53)	36 (64)	0.089
**Double-positives by any method; n (%)**	13 (77)	28 (50)	0.042

**Table 3:** Comparison of prick test results between different ABO blood types.

	A (n = 31)	B (n = 9)	AB (n = 5)	0 (n = 21)	p
Median Apis Mellifera prick diameter (mm)	3 (0–5.25)	0 (0–6)	3 (1–4.5)	3 (0–5)	0.89
Median Vespula diameter (mm)	4 (3–4.5)	3 (1.5–4)	0 (0–4.5)	3 (0–5.5)	0.568

mm: millimeters

**Table 4:** Comparison of prick test results between two Rh blood types.

	Rh+ (n = 60)	Rh-(n = 6)	p
Median Apis Mellifera prick diameter (mm)	3 (0–5)	4 (1.5–5.25)	0.65
Median Vespula prick diameter (mm)	3 (2.25–4.25)	1.5 (0–4.75)	0.372

mm: millimeters

**Table 5:** Demographic and characteristic data of patients who experienced systemic reactions in the maintenance phase.

Patient	Serum Tryptase	Chronic Disease	Autoimmune Disease	Beta Blocker	Ace INHB	Polysensitization	High Dose
Patient 1	>11.4 μg/L	Hepatitis B		−	−	+	−
Patient 2	Normal			−	−	+	−
Patient 3	Normal	−	−	−	−	+	+
Patient 4	>11.4 μg/L	−	−	−	−	+	−
Patient 5	Normal (2.7 μg/L)	−	−	−	−	+	+
Patient 6	−	−	−	−	−	+	−
Patient 7	Normal (4.36 μg/L)	−	−	−	−	−	+
Patient 8	−	−	−	−	−	+	+
Patient 9	Normal (8.6 μg/L)	HT	−	+	−	−	−
Patient 10	−	−	−	−	−	−	−
Patient 11	−	−	−	−	−	−	−

ACE INHB: Angiotensin-converting-enzyme inhibitors, HT: hypertension

**Table 6:** Venom immunotherapy protocols with omalizumab.

Patient 1	Patient 2
Systemic reaction with 4. vial 0.8 cc dose of immunotherapy	Systemic reaction with 4. vial 0.6 cc dose of immunotherapy
week 0: 150 mg omalizumab	week 0: 150 mg omalizumab
week 1: 4. vial 0.3 cc	week 2: 4. vial 0.4 cc (with 150 mg omalizumab before 4 h), no reaction
week 2: 4. vial 0.4 cc (with 150 mg omalizumab before 4 h), no reaction	week 3: 4. vial 0.5 cc, no reaction
week 3: 4. vial 0.5 cc, no reaction	week 4: 4. vial 0.6 cc (with 150 mg omalizumab before 4 h), no reaction
week 4: 4. vial 0.6 cc (with 150 mg omalizumab before 4 h), no reaction	week 5: 4. vial 0.7 cc, no reaction
week 5: 4. vial 0.7 cc, systemic reaction with chills, hypotension, cough and dyspnea	week 6: 4. vial 0.8 cc (with 150 mg omalizumab before 4 hours), no reaction
	week 7: 4. vial 0.9 cc, no reaction
	week 8: 4. vial 1 cc (with 150 mg omalizumab before 4 hours), no reaction
	week 10: 4. vial 1 cc, no reaction
	week 13: 4. vial 1 cc, no reaction
	week 17: 4. vial 1 cc, systemic reaction with facial erythema and itching, nausea, uvula edema and dyspnea
	week 19: 4. vial 0.4 cc (with 150 mg omalizumab before 4 h), systemic reaction with uvula edema, dyspnea, facial erythema and itching

## Data Availability

The datasets used and/or analyzed during the current study are available from the corresponding author on reasonable request.
